# Tracking carbon from subduction to outgassing along the Aleutian-Alaska Volcanic Arc

**DOI:** 10.1126/sciadv.adf3024

**Published:** 2023-06-28

**Authors:** Taryn Lopez, Tobias P. Fischer, Terry Plank, Alberto Malinverno, Andrea L. Rizzo, Daniel J. Rasmussen, Elizabeth Cottrell, Cynthia Werner, Christoph Kern, Deborah Bergfeld, Tehnuka Ilanko, Janine L. Andrys, Katherine A. Kelley

**Affiliations:** ^1^ Geophysical Institute, University of Alaska Fairbanks, Fairbanks, AK, USA.; ^2^ Alaska Volcano Observatory, UAF Geophysical Institute, Fairbanks, AK, USA.; ^3^ University of New Mexico, Albuquerque, NM, USA.; ^4^ Lamont Doherty Earth Observatory, Columbia University, Palisades, NY, USA.; ^5^ Istituto Nazionale di Geofisica e Vulcanologia, Sezione di Milano, Milano, Italy.; ^6^ Department of Earth and Environmental Sciences, University of Milano-Bicocca, Milano, Italy.; ^7^ Department of Mineral Sciences, National Museum of Natural History Smithsonian Institution, Washington, DC, USA.; ^8^ U.S. Geological Survey Contractor, New Plymouth, New Zealand.; ^9^ Cascades Volcano Observatory, U.S. Geological Survey, Vancouver, WA, USA.; ^10^ California Volcano Observatory, U.S. Geological Survey, Moffett Field, CA, USA.; ^11^ University of Waikato, Hamilton, New Zealand.; ^12^ Graduate School of Oceanography, University of Rhode Island, Narragansett, RI, USA.

## Abstract

Subduction transports volatiles between Earth’s mantle, crust, and atmosphere, ultimately creating a habitable Earth. We use isotopes to track carbon from subduction to outgassing along the Aleutian-Alaska Arc. We find substantial along-strike variations in the isotopic composition of volcanic gases, explained by different recycling efficiencies of subducting carbon to the atmosphere via arc volcanism and modulated by subduction character. Fast and cool subduction facilitates recycling of ~43 to 61% sediment-derived organic carbon to the atmosphere through degassing of central Aleutian volcanoes, while slow and warm subduction favors forearc sediment removal, leading to recycling of ~6 to 9% altered oceanic crust carbon to the atmosphere through degassing of western Aleutian volcanoes. These results indicate that less carbon is returned to the deep mantle than previously thought and that subducting organic carbon is not a reliable atmospheric carbon sink over subduction time scales.

## Teaser

Aleutian volcanic gas outputs require modest to substantial recycling of altered oceanic crust and subducted sediment carbon.

## INTRODUCTION

Volatile cycling between Earth’s mantle, crust, and surface reservoirs drives magma generation, volcanism, and the long-term evolution of Earth’s atmosphere and habitability (*1*, *2*). Volatiles can be removed from Earth’s surface reservoirs through processes such as weathering, deposition as marine sediments, accretion onto the upper plate, and subduction into Earth’s crust and mantle. Volatiles trapped in the subducting slab can also be released into the overlying crust and mantle wedge via processes such as devolatilization and melting and recycled back to Earth’s atmosphere through volcanism. The ultimate fate of subducted volatiles is controlled by competing forces related to the physical and chemical characteristics of subduction zones (*3*).

Carbon recycled to arc volcanoes originates from the subducted slab, mantle wedge, and overriding crust. Within the subducted slab, carbon derives from trench-fill (terrigenous) and incoming (typically marine) sediments and the altered oceanic crust (AOC). With few exceptions, these reservoirs have distinct carbon isotopic compositions that can be used, along with mixing models, to track volatile migration among these reservoirs. Specifically, AOC is composed primarily of carbonate (inorganic) carbon and has a well-constrained δ^13^C composition of 0.8 ± 0.5‰ (*4*), while mantle carbon is assumed to be relatively constant at −6.5 ± 2‰ (*5*). In contrast, carbon isotope compositions of subducted slab sediments and the overriding crust can vary widely as they can contain both heavy (~0‰) carbonate and light (~−20‰) sedimentary organic carbon (OC) (*3*). While source δ^13^C compositions can be modified during slab devolatilization and degassing processes, we expect these effects to be minor (see Materials and Methods).

The amount of carbon supplied to arc volcanoes from each potential reservoir can be calculated if the carbon isotopic composition or flux for each reservoir is known and sufficiently distinct. Because of the challenges described above, global carbon recycling budgets based on volcanic gas emissions typically assume that all OC and carbonate derive from the subducted slab, ignore potential contributions of carbon from the upper-plate crust, and do not differentiate between carbonate in subducting sediments and AOC (*6*). Calculations also assume a wide range of isotopic compositions for subducted organic sediments and carbonates (*5*) as there are few studies that provide measurements. These simplifications lead to inaccurate accounting of volatile source contributions to volcanic degassing budgets and result in large uncertainties in global carbon budgets over geologic time (*6*, *7*). Recent studies have highlighted the potential importance of upper-plate crust (*7*) and AOC (*8*, *9*) sources to global volcanic carbon budgets; however, quantitative constraints on their contributions are lacking. An important, unresolved question is: how much subducted carbon, in organic and carbonate form, is recycled back to the atmosphere through volcanism versus subducted to the deep mantle (*3*)? This question has implications for Earth’s atmosphere, as preferential deep subduction of OC could serve as a long-term atmospheric carbon sink (*10*).

The Aleutian-Alaska Arc has two characteristics that make it an ideal location to constrain the fate of subducted carbon. First, it is unique in that the upper-plate crust west of 165°W is presumed to lack carbon sources (*11*, *12*), and new constraints presented here indicate minimal carbonate within subducting sediments. These factors greatly simplify carbon cycling calculations as we can assume that all isotopically heavy carbon released from volcanic outputs originates from AOC, and all isotopically light carbon (<−6.5‰) released from volcanic outputs originates from subducted sediments. These attributes eliminate the ambiguity of carbon source contributions found at most arcs. Second, this arc is notable for significant along-strike variations in physical (*13*–*15*) and chemical (*16*–*18*) characteristics that may modulate volatile cycling. Together, these factors can be used to identify characteristics that facilitate or impede carbon recycling to arc volcanoes, enabling future global predictions on the fate of subducting carbon.

Here, we provide a robust evaluation of subducted carbon inputs and volcanic outputs (as CO_2_) along the Aleutian-Alaska Volcanic Arc to investigate the fate of subducting carbon. We present along-arc constraints on the carbon isotopic composition and flux of subducting sediments and on the carbon and helium (He) isotopic compositions of volcanic outputs, to directly compare inputs to outputs. We apply two mixing models to quantify the relative contribution of each carbon source to volcanic outputs, including (i) the established carbon-He model (*5*) and (ii) a forward model (the carbon-isotope model) that uses the carbon isotope composition of subduction inputs to simulate the composition of volcanic carbon outputs (see Materials and Methods). We consider three sources of carbon recycled to Aleutian-Alaska volcanoes: (i) OC within subducted bulk sediments (SED), including both incoming and trench-fill sections; (ii) carbonate from the subducted AOC, and (iii) carbon from the mantle wedge (M). We then evaluate our results in the context of along-arc variations in subduction character to investigate the physiochemical controls of subduction on carbon cycling. Last, we calculate an updated Aleutian-Alaska volcanic carbon flux and quantify the amount of SED and AOC carbon recycled to these volcanoes.

**Fig. 1.**
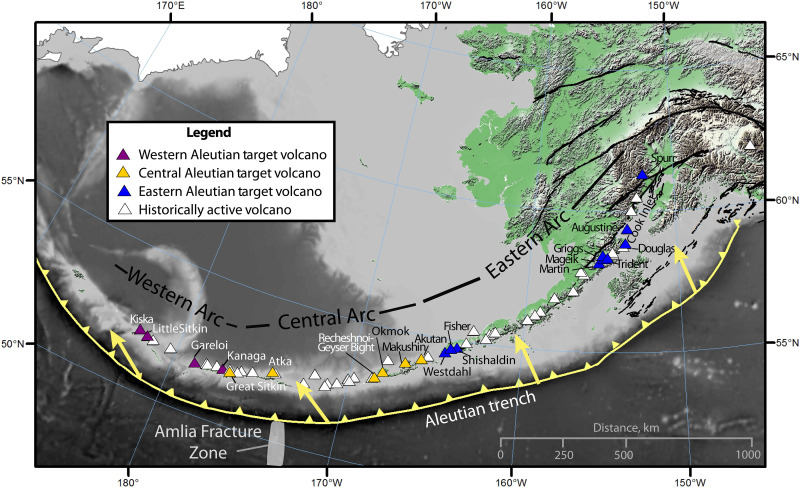
Map of the Aleutian-Alaska Arc. Triangles represent historically active volcanoes, labeled by arc segment, where purple, yellow, and blue colors indicate volcanoes targeted in this study from the western, central, and eastern Aleutian segments, respectively. Arc segments (labeled in black) are defined as the eastern Aleutians (<165°W), including Unimak Island, the Alaska Peninsula, and Cook Inlet region; the central Aleutians from Akun (unlabeled) to Great Sitkin (between 165° to 176.5°W); and the western Aleutians from Adak west (>176.5°W). The approximate trench location and subduction direction are shown in yellow. Figure modified from Buurman *et al.* (*15*).

**Fig. 2.**
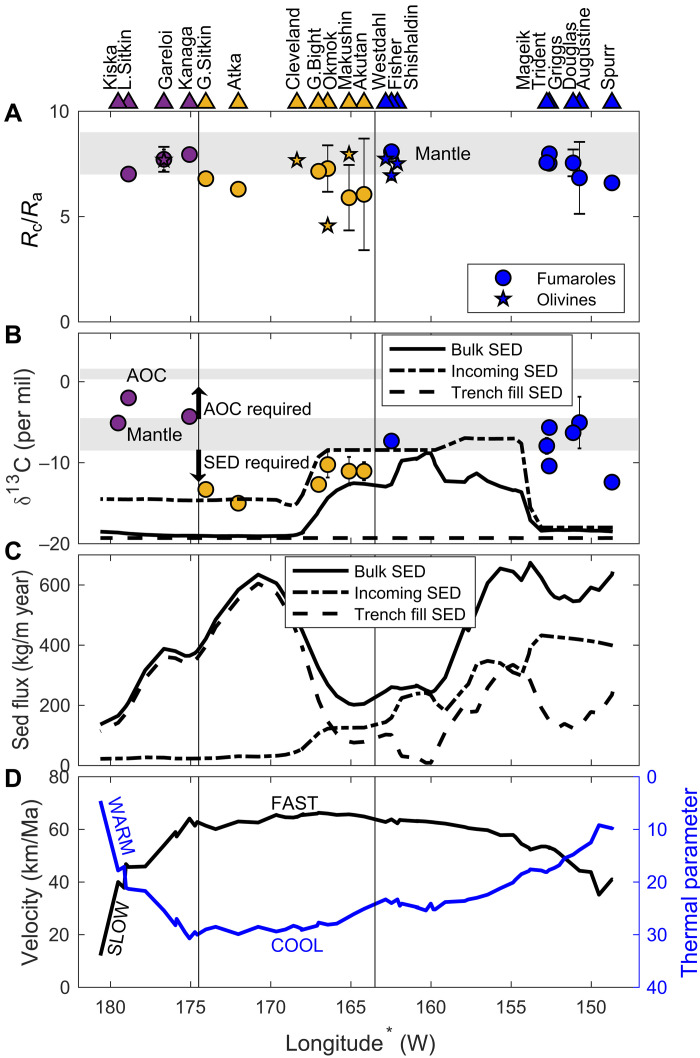
Along-arc variations in subduction inputs, volcanic outputs, and subduction character plotted against projected trench longitude (*). (**A**) Air-corrected helium isotopic compositions for fumarole gas (circles) and olivine-hosted fluids (stars). (**B**) δ^13^C compositions of fumarole gas (circles) compared to that of incoming, trench fill, and bulk sediment (black lines). Error bars represent 1-sigma SD of the measurements for locations with multiple samples. Gray bands indicate the compositional range of M and AOC sources. (**C**) Flux of incoming, trench-fill, and bulk (incoming + trench-fill) sediments entering the Aleutian trench. (**D**) Along-arc variations in trench-normal convergence velocity (black line) and thermal parameter (blue line) (*14*), where smaller values indicate a warmer temperature (*19*). Triangles at top of figure mark the locations of volcanoes with δ^13^C-CO_2_ and/or *R*_c_/*R*_a_ constraints used in this study, colored by arc segment and labeled with names offset slightly in longitude to be legible.

We divide the arc into three segments broadly consistent with those of Kelemen *et al.* (*17*): (i) the eastern Aleutians (longitude <165°W), (ii) the central Aleutians (165° to 176.5°W), and (iii) the western Aleutians (>176.5°W; Fig. 1). The division between the eastern and central Aleutians marks the transition between continental crust to the east and oceanic crust to the west (*13*). This division simplifies our carbon cycling calculations because the eastern Aleutian arc is built on continental crust containing crustal carbon sources (*12*), while the central and western Aleutian segments are built on oceanic crust, where crustal carbon sources are negligible. Our division between the central and western Aleutians is between Great Sitkin and Adak Islands where an abrupt change in carbon isotopic composition of volcanic outputs occurs (Fig. 2). We use these arc segment divisions and data on trench-normal convergence velocity and slab thermal parameter (table S1) (*14*) to characterize along-arc variations in Aleutian-Alaska subduction, where the thermal parameter is the product of trench normal convergence rate and plate age, with smaller values indicating warmer slab temperatures (*19*). Together, these attributes indicate that the eastern and western Aleutian segments are characterized by slow and warm subduction, and the central Aleutian segment is characterized by fast and cool subduction.

## RESULTS

Our volcanic gas dataset includes δ^13^C compositions from 17 volcanic centers, with at least three volcanoes per arc segment. This includes new analyses of volcanic gas data from western and central Aleutian volcanoes and helium isotopic compositions from olivine-hosted fluid inclusions for volcanoes along the arc. Constraints provided by CO_2_/^3^He and δ^13^C for 10 of these volcanoes allow carbon-He model calculations. The volcanic gas outputs show fairly consistent helium-isotopic signatures along strike, while carbon-isotopic compositions show notable variations (Fig. 2 and table S2). Helium isotope ratios of volcanic gases and olivine-hosted fluid inclusions (*R*_c_) are presented relative to air (*R*_a_) (*20*) and mostly have isotope ratios of *R*_c_/*R*_a_ ~7 ± 1, similar to mid-ocean ridge basalts and fluids (8 ± 1) (*20*). Several volcanoes in the central Aleutians have samples with *R*_c_/*R*_a_ < 6 (Fig. 2A and tables S2 and S3) that were primarily collected from volcano flanks, where greater interaction with crustal fluids likely contributed radiogenic ^4^He (*20*). Carbon isotopes in volcanic gases are light (δ^13^C < −10‰) in the central Aleutian and for select eastern Aleutian volcanoes (Fig. 2B), relative to arcs globally (*7*). This isotopically light central Aleutian and eastern Aleutian carbon is consistent with SED-dominated sources. Heavier δ^13^C values (>−6.5‰) in western Aleutian volcanoes indicate predominantly M and/or AOC sources.

Along-strike variations in sediment fluxes entering the Aleutian-Alaska trench and carbon-isotopic compositions for both incoming and trench-fill sections were calculated from sediment thickness, carbon concentrations, and isotope models based on four sites drilled outboard of the Aleutian-Alaska trench (Fig. 2, B and C; fig. S1; and table S1). The SED composition comprises both incoming and trench-fill components. Carbon isotopic compositions of incoming sediments are light, ranging from <−14‰ in the eastern and western Aleutians to −8‰ in the central Aleutians (Fig. 2B). Trench-fill sediments consist of terrigenous turbidites with low δ^13^C values estimated at ~−19‰ along the arc. The bulk sediment carbon isotopic composition ranges from a minimum of ~−19‰ throughout much of the arc to heavier values in the eastern central Aleutians and western eastern Aleutians regions, with a maximum of −8‰ near ~160°W (Fig. 2B and table S1). The flux of incoming sediment entering the Aleutian trench declines from east to west, while bulk sediment fluxes are variable, showing two maxima at ~155°W (eastern Aleutians) and ~171°W (central Aleutians) and a minimum at ~165°W (central Aleutians; Fig. 2C).

**Fig. 3.**
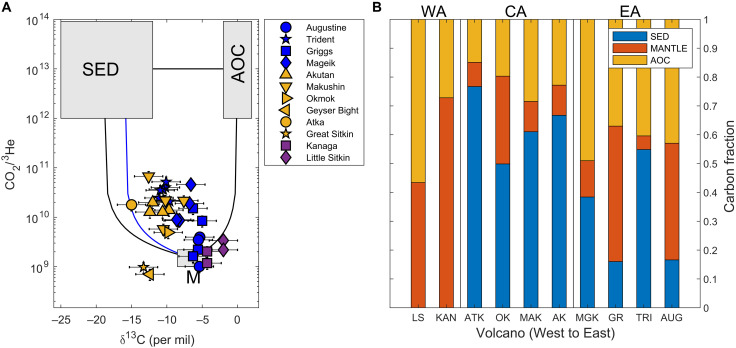
Carbon-He mixing model results for the Aleutian Arc. (**A**) Volcanic gas compositions relative to those of end-member carbon sources of bulk sediment (SED), carbonate presumed to be from the AOC, and mantle (M) following Sano and Marty (*5*). Volcano symbols are colored by arc segment, where purple, yellow, and blue indicate western, central, and eastern Aleutian segments, respectively. The black M-SED line shows two-component M-SED mixing for the arc-minimum SED δ^13^C value (−19‰), while the blue line shows the M-SED mixing line for the minimum SED δ^13^C value (−16‰) for CA volcanic centers of Akutan, Makushin, Okmok, and Geyser Bight, which have a notably heavier SED δ^13^C compositions (see Fig. 2). Error bars represent ±25% on the *y* axis and ± 2‰ on the *x* axis to capture chemical and isotopic fractionation due to degassing, which are both larger than the analytical uncertainties in the sample analysis. (**B**) Normalized mean proportions of SED, AOC, and M carbon sources based on calculations for Aleutian-Alaska volcanoes shown in (A) from West to East: Little Sitkin (LS), Kanaga (KAN), Atka (ATK), Okmok (OK), Makushin (MAK), Akutan (AK), Mageik (MGK), Griggs (GR), Trident (TRI), and Augustine (AUG). Arc segments labeled above by WA for western Aleutians, CA for central Aleutians, and EA for eastern Aleutians, with vertical lines marking division.

**Fig. 4.**
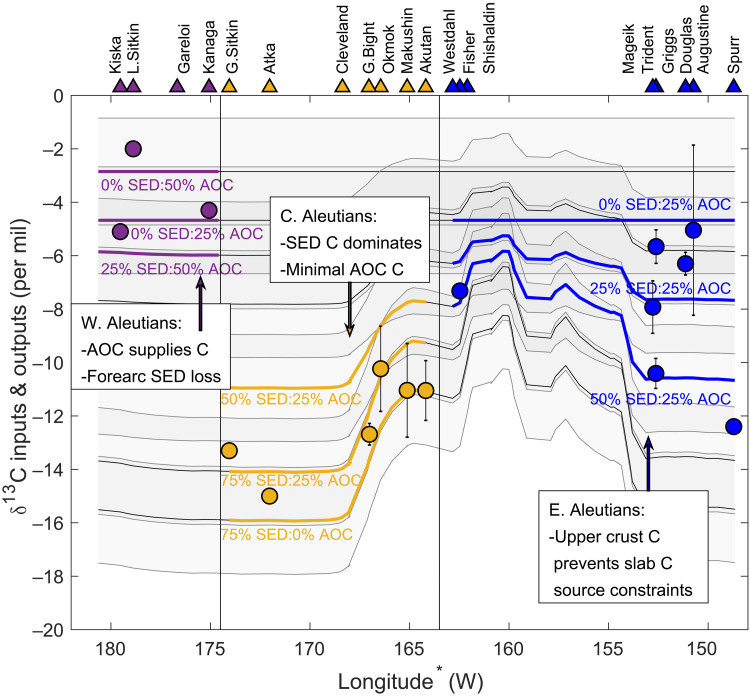
Preferred carbon-isotope model results per arc segment. Observed volcanic δ^13^C-CO_2_ outputs from fumarole samples (filled circles) and select predicted volcanic carbon outputs calculated from the carbon-isotope model as mixtures of M, SED, and AOC (lines). Each line shows the percent of carbon (C) supplied from bulk sediment (SED) for a scenario in fig. S3, with the remaining carbon being attributed to mantle (M) and/or AOC sources. Blue, yellow, and purple lines show examples of preferred carbon-isotope model results to explain volcanic gas outputs for the eastern, central, and western Aleutians, respectively. Shaded regions reflect ±2‰ uncertainties for each model. The key findings related to carbon recycling to volcanoes within each arc segment are summarized in text boxes.

The carbon-He and carbon-isotope model results show the proportion of volatiles originating from M, AOC, and SED sources supplied to each volcano (Figs. 3 and 4, figs. S3 and S4, and tables S1 and S2). Several samples fall outside the mixing boundaries in Fig. 3, likely due to preferential loss of CO_2_ relative to ^3^He during volatile exsolution and degassing and/or due to partial dissolution of CO_2_ in near-surface waters that can lead to calcite precipitation (*21*, *7*). These samples are normalized to their two dominant sources. The proportions of M, AOC, and SED vary along the arc, with both models indicating notable commonalities within each arc segment. The eastern Aleutian model outputs show variable mixtures of carbon sources over a small region (Figs. 3B and 4). The high variability seen over small spatial scales (e.g., for neighboring volcanoes Mageik, Griggs, and Trident) is consistent with discrete contributions of coal-bearing sedimentary units from the overriding continental crust (*11*, *12*). Because the eastern Aleutian data do not allow us to uniquely quantify deep carbon sources (mantle and slab), we focus our approach and interpretations on the central and western Aleutian arc segments.

The central Aleutian volcanic outputs reflect a substantially greater proportion of SED-derived carbon relative to M and AOC sources, as seen in both model results. The light δ^13^C signatures of central Aleutian volcanic outputs are explained by >50% of carbon supplied by subducted SED, with the remainder (<50%) from M and AOC sources (Figs. 3 and 4, figs. S3 and S4, and tables S2 and S4). In contrast, the heavier δ^13^C composition of western Aleutian volcanic outputs originates primarily from M and AOC (>75%), with minimal (<25%) to no carbon from subducted SED sources (Figs. 3 and 4, figs. S3 and S4, and tables S2 and S4).

The most marked change in the carbon-isotopic composition of volcanic outputs occurs between Kanaga and Great Sitkin volcanoes, at the central-western Aleutian transition (Fig. 2B). The high proportion of SED-derived carbon supplied to central Aleutian volcanoes is in stark contrast to the western Aleutians, where volcanic carbon emissions are supplied primarily by M and AOC (Fig. 3B). This transition cannot be explained simply by variations in sediment entering the trench, as SED fluxes are similar for both central-western Aleutian segments (Fig. 2).

## DISCUSSION

We propose that the marked differences in carbon source contributions to central and western Aleutian volcanoes result from preferential sediment subduction in the central Aleutians and preferential forearc sediment removal in the western Aleutians, as modulated by subduction character. The central Aleutian arc segment is characterized by perpendicular convergence, relatively fast subduction, and a relatively cool slab temperature (Fig. 2D) (*14*), characteristics that favor sediment subduction (*22*). We infer that in the central Aleutians, fast and cool subduction facilitates sediment subduction to sub-arc depths and recycling of SED carbon to volcanic outputs. In contrast, the western Aleutian arc segment is characterized by relatively slow and oblique convergence and a warmer slab temperature (Fig. 2D) (*14*) that leads to shallow (i.e., forearc) removal of subducting sediments through accretion/underplating, devolatilization, and/or melting/diapirism (*22*–*24*), with minimal SED recycled to western Aleutian volcanoes. We speculate that both sediment accretion/underplating and devolatilization/melting are occurring in the western Aleutian forearc, with the former varying on a local-scale due to tectonic controls, such as plate coupling, and the latter being largely responsible for the regional-scale difference between the central and western Aleutian segments. Variations in plate coupling from high (near Kanaga) to low (near Great Sitkin) could explain the abrupt change in carbon source contributions at the central-western Aleutian boundary (*25*). While we cannot differentiate between the processes of sediment accretion/underplating and devolatilization/melting within the forearc, the outcome is the same: Less sedimentary carbon reaches sub-arc depths in the western Aleutians, relative to the central Aleutians. These interpretations are supported by previous studies that found preferential recycling of subducted sediments to central Aleutian volcanoes and an increase in AOC (as eclogite) to western Aleutian volcanoes based on along-arc variations in the chemistry of erupted lavas (*16*–*18*). Furthermore, retention of sedimentary carbon to sub-arc depths is consistent with recent thermal models for central Aleutian volcanoes (*26*). These combined findings suggest that variations in physical subduction character can lead to substantial differences in slab-derived carbon recycling efficiencies to arc volcanoes.

We use our constraints on subducted carbon inputs, proportion of carbon recycled from SED and AOC sources, and updated volcanic CO_2_ fluxes modified from (*27*) to calculate a carbon cycling budget for the Aleutian-Alaska Arc (see Materials and Methods; tables S5 and S6). We find a total Aleutian-Alaska volcanic carbon output of 0.455 ± 0.316 Tg C/year and a total slab (SED + AOC) input of ~2.107 Tg C/year (table S6). On an arc-wide scale (excluding the crustally contaminated eastern Aleutian), we estimate that ~16% of subducted slab carbon is recycled to the atmosphere via arc volcanism (table S6). On a regional scale, we estimate that ~5 to 6% of subducted AOC carbon is recycled to both western and central Aleutian volcanoes, while 0 and ~43% of SED carbon is recycled to western Aleutian and central Aleutian volcanoes, respectively (table S6). If we consider an end-member scenario in which only incoming sediments are subducted to sub-arc depths (i.e., all trench-fill sediments are lost to shallow processes), then the proportion of SED relative to AOC required to explain volcanic outputs increases, and recycling of AOC to central Aleutian volcanoes is not required. Collectively, the most likely and end-member scenarios find that ~43 to 61% of SED is recycled to central Aleutian volcanoes with absolute upper and lower bounds of 3 to 100%, while the most likely percentage of AOC recycled to western Aleutian volcanoes is ~6 to 9%, with absolute upper and lower bounds of 1 to 34% (table S6). These calculations emphasize that modest but quantifiable recycling of both SED and AOC carbon are required to explain Aleutian-Alaska volcanic gas signatures, even if all trench-fill sediments are lost to forearc processes.

The modest recycling of AOC carbon to western Aleutian (and likely central Aleutian) volcanoes confirms recent findings that the subducted slab AOC is an important source of volcanic carbon emissions to the atmosphere (*8*, *9*) and suggests that previous estimates of AOC carbon return to the mantle may be overestimated. While volcanic gas studies have long inferred recycling of subducted AOC carbon to arc volcanoes (*5*), it has been difficult to confirm since most arcs have multiple carbonate sources. In addition, previous studies indicate that AOC carbon is largely returned to the mantle (*28*) but can be released at sub-arc or shallower depths under favorable conditions (*9*, *19*, *26*, *29*). Our findings suggest instead that modest recycling of subducted AOC carbon may be the norm rather than the exception.

Last, we find that substantial subducted SED OC can be efficiently recycled to the atmosphere under favorable subduction conditions. This result challenges previous studies’ conclusions that subducted OC is preferentially sequestered in the slab and transported to the deep mantle (*3*, *10*). Instead, we find that substantial subducted OC is returned to Earth’s surface over subduction time scales and that subducting OC is not a reliable carbon sink for Earth’s atmosphere.

## MATERIALS AND METHODS

### Modeling

We use two approaches to constrain the proportions of three carbon sources that contribute to volcanic carbon outputs along the Aleutian-Alaska Arc. Throughout, we assume that all carbon is released as CO_2_. The carbon-He model is the standard three-component mixing model of Sano and Marty (*5*) that uses the CO_2_/^3^He and δ^13^C-CO_2_ composition of volcanic gases (table S2) and assumes end-member compositions for mantle (M), bulk organic sediment (SED), and carbonate (AOC) to calculate the proportion of volatiles supplied by each source. The second model, the carbon-isotope model, is a forward model that uses the carbon isotope composition of possible source inputs and mass balance, to explain the isotope composition of volcanic outputs (*C*_v_) following Eqs. 1 and 2

$$C_{v}\approx C_{\mathrm{SED}+M+\mathrm{AOC}}=(C_{\mathrm{SED}}\times f_{\mathrm{SED}})+(C_{M}\times f_{M})+(C_{\mathrm{AOC}}\times f_{\mathrm{AOC}})$$ (1)

$$f_{\mathrm{SED}}+f_{M}+f_{\mathrm{AOC}}=1$$ (2)

where *C* represents the δ^13^C composition, and *f*_SED_, *f*_M_, and *f*_AOC_ represent the fraction of each of the three sources contributing to volcanic outputs. For both models, we assume that organic sediment and carbonate are supplied entirely from subducted sediments and the AOC, respectively. The latter is a valid assumption for central and western Aleutian volcanoes, where crustal carbon is thought to be negligible. We acknowledge that crustal carbon, in both organic and carbonate forms, may be contributing to volcanic outputs in the eastern Aleutians. Both the carbon-He model and the carbon-isotope model assume a M δ^13^C composition of −6.5 ± 2‰ (*5*) and an AOC δ^13^C composition of 0.8 ± 0.5‰ (*4*). The carbon-He model uses the observed arc minimum subducted SED δ^13^C input of −19 ± 1‰ for all volcanoes, except the central Aleutian volcanic centers of Akutan, Makushin, Okmok, and Geyser Bight, where we use the minimum observed segment SED δ^13^C value of −16 ± 1‰ (Fig. 2B and table S1), thus providing minimum estimates of the SED proportion. The carbon-isotope model uses the actual SED δ^13^C composition of the bulk sediments, estimated along-strike and projected to each arc volcano (table S1). The carbon-He model uses a mantle CO_2_/^3^He composition of 1.67 ± 0.21 × 10^9^ (*20*) and a value of 1 × 10^13^ (range from 10^12^ to 10^14^) for both SED and AOC carbon (*5*).

The carbon-He model has the advantage of enabling volatile source proportions to be calculated using only volcanic gas data and general constraints on end-member source compositions. The main disadvantage of this model is that CO_2_ and He concentrations, as well as δ^13^C compositions, may be fractionated by degassing or modified by other nonsource processes (*30*). Recent work by Tucker *et al.* (*21*) suggests that open-system degassing will decrease the CO_2_/^3^He ratio in mid-ocean ridge basalt by less than 25% in most cases; therefore, we use ±25% as an upper limit to our uncertainties in CO_2_/^3^He for our volcanic gas outputs to account for degassing-driven chemical fractionation, which is expected to be much larger than the analytical uncertainty.

The carbon-isotope model has the advantage that it does not require constraints on the volcanic gas ^3^He/^4^He composition and thus on ^3^He concentration, which, in the case of the Aleutian-Alaska Arc, allows use of a larger dataset with broader spatial coverage. This model also takes advantage of improved constraints on along-arc variations in δ^13^C composition and the presumed flux of subducted sediment inputs. The disadvantage of this approach is that Eqs. 1 and 2 together have three unknowns (*f*_SED_, *f*_M_, and *f*_AOC_). To solve these equations, we consider a range of AOC carbon contributions to volcanic outputs of 0, 25, 50, and 75%, with the remaining carbon supplied from a mixture of SED and M sources, which are varied by 25% increments.

One further concern with both models is the potential for modification of the initial source δ^13^C composition between the onset of subduction and throughout outgassing. The main process that could modify the δ^13^C composition is isotopic fractionation that can occur during slab devolatilization or during magma degassing. Both occurrences would lead to CO_2_ gas being enriched in δ^13^C, leaving a lighter solid slab and/or fluid melt depleted in δ^13^C (*31*, *32*). If the processes are completed in a closed system and allowed to equilibrate, there will likely be no obvious modification to the initial δ^13^C composition (*7*). If these processes occur in an open system, where the gas and solid/liquid are immediately separated from each other, continued degassing of CO_2_ would lead to a shift toward lighter δ^13^C-CO_2_ as the magma/slab is continually depleted in δ^13^C (*33*). Previous observational studies on both exhumed subduction sections and volcanic CO_2_ emissions suggest that isotopic fractionation due to slab decarbonation can be considered negligible [e.g., (*5*, *34*–*36*)]. Similarly, volcanic observations of temporal variations in δ^13^C-CO_2_ over a volcanic eruption/unrest cycle suggest that the shift in δ^13^C within volcanic gases due to magmatic outgassing is limited to ~2‰ (*37*, *38*). More recently Tumiati *et al.* (*39*) proposed that the δ^13^C-CO_2_ compositions of volcanic gases reflect the CO_2_/CaCO_3_ of the slab for systems containing both oxidized carbon (aragonite) and reduced carbon (graphite). Because the incoming Aleutian sediments contain no carbonate, and trench-fill Aleutian sediments only contain rare carbonate (see subducting sediment input section below), we expect the proposed CO_2_/CaCO_3_ buffering mechanism to exert minimal influence on the Aleutian-Alaska volcanic δ^13^C-CO_2_ compositions. Considering the above, we assume modification from the source δ^13^C-CO_2_ by CO_2_/CaCO_3_ buffering to be negligible and by isotopic fractionation due to slab devolatilization and magma degassing to be limited to ±2‰ and use this value as our uncertainty.

To directly compare volcanic gas outputs to subducted inputs, we use volcano-specific projected trench longitudes [calculated from the North America-Pacific Euler pole by DeMets *et al.* (*40*)] and assume that the sediment composition and flux entering the trench today is similar to what was supplied in the past (i.e., steady-state conditions over the past few million years). To identify along-arc trends in carbon sources using the carbon-He model, we calculate the mean contribution of the three volatile sources to each individual volcano. Several samples fall outside the mixing bounds of Fig. 3A, with CO_2_/^3^He values less than the assumed mantle value (~1.67 × 10^9^) (*5*). This is likely a result of preferential loss of CO_2_ relative to ^3^He during volatile exsolution and degassing, due to their different solubilities and diffusivities in basaltic magma; however, it may also reflect heterogeneities in the mantle composition (*21*, *41*). Where values fall outside the mixing bounds, they are normalized to their two dominant sources where possible (e.g., Kanaga and Little Sitkin) or excluded from analyses (e.g., Geyser Bight and Great Sitkin; Fig. 3B and table S2). For the carbon-isotope model, we identify a range of possible M-AOC-SED mixtures that can explain the observed volcanic output δ^13^C value (within error) for at least three volcanoes within each arc segment, including the heaviest values in the western Aleutians and requiring a fit to the three lightest values for the central Aleutians, to ensure that the model can explain these end-members. For the carbon-isotope model, we also conduct calculations assuming that SED is composed of incoming sediments only (with heavier δ^13^C signatures relative to the bulk sediment composition) that may be expected if trench-fill sediments are preferentially accreted/underplated onto the upper plate (fig. S4). This change has only a minor influence on the results, resulting in a slightly higher proportion of subducted SED and slightly lower proportion of M and AOC carbon contributing to eastern and central Aleutian volcanoes, with minimal impact on western Aleutian volcanic outputs (table S4). We exclude two spurious samples from the literature from our mixing calculations. Specifically, Augustine sample ff2 from Motyka *et al.* (*42*) has a significantly heavier δ^13^C composition of (+) 2.4‰ compared to the other Augustine samples (average of −6.1 ± 1.1‰; table S2). We suspect that this is due to a transcription error. The second sample excluded is the Okmok Cone A sample, also from Motyka *et al.* (*42*). This sample is substantially air-contaminated, such that the uncertainty in the composition data seen in table S2 is likely much greater than the other presented samples. The results for the carbon-isotope and carbon-He models are presented in tables S1 and S2, Fig. 3, and figs. S3 and S4 and summarized in Fig. 4 and table S4.

### Volcanic gas measurements

Volcanic gas chemical and isotopic compositions presented here represent results from prior studies (*42*–*48*) and sample analyses following collection during 2015 field campaigns to the central and western Aleutian arc segments (table S2) (*49*, *50*). Samples are from fumaroles or steaming ground with temperatures of boiling point (at their elevation) or greater. Both flank and summit/crater degassing sites are included in the analyses to maximize the dataset. However, we acknowledge that gas from flank fumaroles may have a greater influence of shallow (non-magmatic) processes (table S2). Samples from springs or other subaqueous degassing manifestations are not considered to minimize potential effects of isotope fractionation. Where multiple samples from the same collection site and date are available, the sample containing the largest number of relevant gas species measured (e.g., CO_2_, He, δ^13^C-CO_2_, and *R*_c_/*R*_a_) is reported here. Often, each sample did not contain analyses for all relevant gas species. In these cases, data from duplicate samples are reported to provide complete analyses of carbon and He. These are reported in the “notes” column of table S2. In several cases, samples reported in the literature [e.g., data from (*42*–*44*)] did not have unique sample numbers to allow us to report gas and isotopic compositions. These data from ambiguous samples were excluded from analysis and are not reported in table S2.

The 2015 central Aleutian gas samples were collected and analyzed following the methods of Bergfeld *et al.* (*45*). Gases were collected directly from fumarolic vents or steaming ground into evacuated bottles for CO_2_, He, and δ^13^C-CO_2_ analyses and into copper tubes for ^3^He/^4^He analyses. Complete details on the sample collection and analyses methods for 2015 central Aleutian samples can be found in Bergfeld *et al.* (*45*) and Werner *et al.* (*49*).

The 2015 western Aleutian gas samples were collected from fumarolic vents into evacuated bottles containing a caustic (4 M NaOH) solution for CO_2_ and He concentrations and in copper tubes for ^3^He/^4^He ratios following the methods of Giggenbach and Gougel (*51*). These samples were analyzed for CO_2_ and He at the University of New Mexico following the methods in (*52*) and for ^3^He/^4^He at the Istituto Nazionale di Geofisica e Vulcanologia (INGV) Sezione di Palermo (Italy) following the methods described by Rizzo *et al.* (*53*). In addition, for the western Aleutian volcanoes of Kiska, Little Sitkin, and Kanaga, between five and eight plume samples, representing a mixture of volcanic gas and ambient air in varying concentrations, were collected within ~2 m of individual or coalesced fumarole vents into Tedlar bags. These samples were analyzed in the field for δ^13^C-CO_2_ and CO_2_ mixing ratios using a Delta Ray Isotope Ratio Infrared Spectrometer following the methods of Fischer and Lopez (*54*). Results from δ^13^C-CO_2_ compositions and CO_2_ concentrations of the plume samples and ambient air were used to linearly extrapolate the original (i.e., volcanic) δ^13^C-CO_2_ compositions from these volcanoes (fig. S2). Fisher and Lopez (*54*) compared the extrapolated plume δ^13^C-CO_2_ composition to that derived from a direct fumarole sample analyzed using traditional methods for Kanaga volcano and found the results to be within error; therefore, we assume that these results are comparable for the other western Aleutian volcanoes. For some 2015 western Aleutian samples, duplicate samples were collected for gas CO_2_ and He compositions from the same or neighboring fumaroles, but isotope analyses were only conducted for the bulk plume. In such cases, the bulk δ^13^C-CO_2_ composition was applied to all samples for a given site.

### He isotope measurements in olivine-hosted fluid inclusions

Tephra samples that were processed and analyzed for olivine-hosted fluid inclusions were collected during two different GeoPRISMS campaigns that took place during the 2015 field season (table S3). The first campaign visited the eastern and central Aleutian volcanoes of Shishaldin, Fisher, Westdahl, Makushin, Okmok, and Cleveland. The samples are basaltic ash and lapilli tephras. Samples from Fisher, Shishaldin, Okmok, and Cleveland were collected on cinder cones. Westdahl samples were collected on a volcanic ridge on the southeast flank of Westdahl. The sample from Makushin was collected on Pakushin, a composite cone located on Makushin’s southwest flank. Samples were washed in tap water, dried, and sieved into size fractions. Lithium heteropolytungstates in water (LST) heavy liquid separation was performed on the 0.5- to 0.1-mm-size fraction. Samples were then washed with deionized water and dried overnight in an oven at 50°C. Olivines (0.5 to 1 g per sample) were handpicked from the heavy fraction and placed into mineral oil. Targeted olivine grains were free of attached matrix glass and with minimal alteration. Most had a low concentration of crystal or melt inclusions. Olivines were then cleaned with soap and water and placed in an ultrasonic bath for two 30-min rounds of cleaning with deionized water.

Basaltic tephra deposits from Gareloi volcano in the western Aleutian segment were collected during the second campaign. Details about field collection methods can be found in the cruise report (*55*). Tephra sample 15GREC009-2 was washed in dilute HCl and deionized water, dried, and sieved into size fractions. LST heavy liquid separation was performed on the 0.5- to 1-mm-size fraction. The sample was then washed again with deionized water and dried. Olivines were handpicked in glycerin from the “heavy” fraction, washed once more in deionized water, and dried under a heat lamp.

Before analysis at the INGV Sezione di Palermo (Italy), all olivines were given a final cleaning in diluted acid (6% HNO_3_) and washed with deionized water and pure acetone in an ultrasonic bath. Seven aliquots of crystals with a weight between 0.1 and 0.7 g were loaded into a stainless-steel crusher that was baked under pumping conditions for 48 to 72 hours at 130°C until ultrahigh vacuum conditions (10^−9^ mbar) were reached. Olivines were then crushed at room temperature (21°C) via the single-step procedure to release gases trapped in fluid inclusions while minimizing the contribution by cosmogenic ^3^He and radiogenic ^4^He trapped in the crystal lattice. Gas species that expanded in the crusher were then purified and analyzed for He, Ne, and Ar isotopic ratios following the analytical technique described by Rizzo *et al.* (*56*, *57*).

### Subducting sediment inputs

Sediment thicknesses along the Aleutian-Alaska trench were taken from Ryan *et al.* (*58*) by digitizing contours in their figure 13 and then averaging over a Gaussian sliding window with a width of ~250 km. For regions of the trench not covered by Ryan *et al.* (*58*), thicknesses were estimated from the GlobSed compilation (*59*). Note that we distinguish the sedimentary section on the incoming plate outboard of the trench, the “incoming sediment,” from that deposited in the trench as “trench-fill.” Sediment densities and carbon concentrations were taken from shipboard analyses reported for drill sites ODP 866 [44.589730°N, 168.240267°W; (*60*)], DSDP 183 [52.571667°N, 161.205500°W; (*61*)], and IODP U1417 [56.959993°N, 147.109975°W; (*62*)]. These were used to calculate the mass-weighted average concentrations of OC and inorganic carbon (IC) at eight reference nodes along the Aleutian-Alaska trench (fig. S1). Values for the carbon isotopic compositions and fluxes were interpolated between nodes at points along the trench that represent the back-projected positions of each volcano. For incoming sedimentary carbon, isotopic compositions were averaged from the global compilation of Hayes *et al.* (*63*) at appropriate age intervals. The carbon isotopes were then weighted by the carbon concentration and thickness of each unit to calculate bulk isotopic compositions for OC and IC separately. The bulk carbon for each node was then calculated by weighting the bulk OC and IC δ^13^C by the OC and IC concentrations and layer thicknesses. The trench-fill derives from the east by downslope transport (fig. S1), and so, its composition is approximated by turbidites cored in the Surveyor Fan section of IODP 1417 in the Gulf of Alaska. Likewise, the δ^13^C of the OC in the trench-fill taken from the OC-weighted mean isotopic composition of sediments from 283 to 700 mbsf in Hole U1417 (*64*) is −26‰, typical of terrestrial carbon. The calculated δ^13^C of the bulk trench-fill is heavier (−19.3‰), after including minor cement carbonate with marine compositions, again, based on the Surveyor Fan section at IODP 1417.

### Sediment thickness and stratigraphy

A large gradient exists in the thickness of the incoming sediment section along strike of the trench (fig. S1). West of 166.5°W (nodes 1 to 3 in fig. S1), a very thin sedimentary section approaches the trench, consisting of pelagic clay and diatom ooze that is carbonate-free due to deposition below the carbonate compensation depth. In the region between 158° and 154°W (nodes 5 to 7 in fig. S1), the seafloor carries the buried Eocene Zodiac Fan, which has been transported north from its formation as far south as the coast of British Columbia. DSDP Site 183 penetrated through the turbidites of the Zodiac Fan, beneath a sequence of pelagic diatom ooze, green clay, and minor carbonate. East of 154°W (nodes 7 to 8 in fig. S1), the seafloor carries turbidites of the Surveyor Fan, derived from the Gulf of Alaska, beneath a Plio-Plestoocene section of gray mud with ice-rafted material. The turbidites were deposited on top of a thin pelagic interval of brown clay and minor carbonate. In general, carbonate sediments are scarce, while organic-carbon bearing terrigenous sediments abound in the Aleutian-Alaska trench.

### Sediment-derived carbon fluxes and isotopic compositions

We quantify incoming sediment fluxes as those on the downgoing plate 100 km outboard of the trench axis and bulk sediment fluxes as those that include the trench-fill. In the central to western Aleutian regions (west of −168°), OC fluxes dominate because the incoming sediment contains no carbonate. Likewise, the trench-fill contains more OC (redeposited terrestrial OC) than carbonate (present only as rare cement within turbidites units). This leads to δ^13^C that is very light, ~−15‰ for the incoming sediment and shifted to even lighter δ^13^C (~−19‰) when including the trench-fill (Fig. 2B). In the central part of the trench (−168° to −154°), the proportion of IC increases because of the appearance of thin pelagic carbonate units. The minimum in trench-fill in this region also leads to a reduction in the potential OC input to the trench. These two effects together lead to a rise in the δ^13^C in the sediments that subduct in the central part of the trench (Fig. 2B). In the eastern part of the trench (east of −154°), OC dominates once again due to the predominance of terrigenous turbidites of the Surveyor Fan, with an isotopic shift to lighter δ^13^C.

While we have well-constrained values on the flux of incoming and trench-fill sediments expected to enter the Aleutian-Alaska trench, we are less certain how much sediment is actually subducted to sub-arc depths versus accreted or underplated onto the upper plate. It is generally assumed that at accretionary margins, such as the Aleutian-Alaska Arc, most incoming sediments are subducted, while most trench-fill sediments are accreted (*65*). Unfortunately, to our knowledge, there are no robust constraints on the amount of incoming and trench-fill sediments accreted along the Aleutian-Alaska Arc. Because the mean measured δ^13^C compositions of volcanic gases in the central Aleutian sector are isotopically lighter than that of the incoming sediments (and sediments are the isotopically lightest carbon source end-member; Fig. 2B), this infers that recycling of at least some trench-fill sediment is required in this sector and potentially throughout the arc. We therefore assume that on an arc-wide scale, all incoming sediments and at least some trench-fill sediments are subducted. We consider the incoming sediment flux to represent the minimum subducted sediment flux and the bulk sediment flux to represent the maximum subducted sediment flux and that the average of these two fluxes represents what is mostly likely subducted. We present the average (i.e., most likely) flux, with the minimum to maximum ranges provided parenthetically in the following. We find average subducted sediment carbon fluxes of 406 (329 to 483), 245 (68 to 423), and 156 (15 to 296) kg C/m year for the eastern, central, and western Aleutian segments, respectively (table S6). When these values are multiplied by the trench segment lengths of 1030 ± 20 (eastern Aleutians), 820 ± 20 (central Aleutians), and 480 ± 20 (western Aleutians) km measured in Google Earth using the projected trench longitude of the first and last volcanoes in each arc segment and converted to Tg C/year, we find subducted sediment inputs of 0.418 (0.332 to 0.508), 0.201 (0.054 to 0.355), and 0.075 (0.007 to 0.148) Tg C/year for the eastern, central, and western Aleutian segments, respectively. This yields a total sediment-derived Aleutian-Alaska carbon input of 0.694 (0.393 to 1.011) Tg C/year (table S6).

### AOC carbon fluxes

Because our observations indicate that the AOC is contributing carbon to volcanic outputs, we calculate a total Aleutian-Alaska AOC carbon input flux. This flux combined with the sediment input flux (above) and volcanic output flux (below) enables us to quantify carbon recycling throughout the Aleutian-Alaska Arc. For the AOC carbon flux calculation, we assume an average AOC carbon concentration of 500 ± 100 parts per million (*3*), a slab crust thickness of 7100 ± 800 m (*66*), and a slab crust density of 2970 ± 20 kg/m^3^ (*67*) for the entire arc. These parameters are multiplied by arc segment–specific arc lengths (above) and trench-normal convergence rates (table S1) (*14*) to find total AOC carbon fluxes of 0.602 (0.394 to 0.868), 0.559 (0.373 to 0.792), and 0.253 (0.145 to 0.406) Tg C/year for the eastern, central, and western Aleutian arc segments, respectively. This yields a total Aleutian-Alaska AOC carbon input flux of 1.413 (0.912 to 2.065) Tg C/year (table S6) and a total SED + AOC flux of carbon into the Aleutian-Alaska trench of ~2.107 (1.306 to 3.076) Tg C/year (table S6).

### Volcanic carbon output fluxes

CO_2_ fluxes from Aleutian-Alaska volcanoes were calculated in Fischer *et al.*, (*27*) and are updated here based recent activity through 2022 and/or knowledge of eruptive activity over the study period (2005 to 2022) from the Alaska Volcano Observatory (*68*). Since the majority of volcanic gases are emitted quiescently during noneruptive periods (*69*), and these emissions best represent long-term degassing trends, we focus here on passive CO_2_ emissions. We use CO_2_ emission estimates compiled by Fischer *et al.* (*27*) in most cases. Following these methods, CO_2_ emission rates are extrapolated for degassing volcanoes characterized as either magmatic or hydrothermal when no measurements are available. Fischer *et al.* (*27*) classify magmatic degassing volcanoes as those that produce a coherent, fumarolic plume, and/or have undergone eruption during the study period. They classify hydrothermal degassing volcanoes as those who release gases through water, mud, and/or steaming ground and have no coherent plume or large fumaroles. Fischer *et al.* (*27*) use a magmatic extrapolation value of 0.16 Tg CO_2_/year and a hydrothermal extrapolation value of 0.013 Tg CO_2_/year based on statistical analysis of a global compilation of “weakly” degassing volcanoes. We first update the classification of Aleutian-Alaska volcanoes from Fischer *et al.* (*27*) that underwent eruption between 2005 and 2022 but do not have CO_2_ measurements to use the magmatic extrapolation value. These volcanoes include Pavlof, Okmok, Kasatochi, Great Sitkin, and Semisopochnoi. We then evaluated the global extrapolation values used in Fischer *et al.* (*27*) to see whether they are appropriate for use in Alaska. Of the 71 Aleutian-Alaska volcanoes considered in Fischer *et al.* (*27*), 40 were identified as degassing, and 17 have reported measured CO_2_ fluxes. Of these 17 measured volcanoes, none have CO_2_ fluxes that exceed the magmatic extrapolation value used in Fischer *et al.* (*27*). We therefore conclude that Aleutian-Alaska volcanoes have lower than global average magmatic CO_2_ fluxes and instead use a magmatic extrapolation value equal to the mean ± SD of measured “magmatic” Alaska volcanoes calculated from Fischer *et al.* (*27*) of 0.0723 ± 0.063 Tg CO_2_/year. We also evaluate the hydrothermal extrapolation value (0.013 ± 0.005 Tg CO_2_/year) used in Fischer *et al.* (*27*) and find that this value agrees within error with the single Aleutian-Alaska measured CO_2_ diffuse degassing flux from Ukinrek-Mars by Evans *et al.* (*70*) of 0.068 ± 0.053 Tg CO_2_/year and use the value from Fischer *et al.* (*27*) for hydrothermal volcanoes. Summing the volcanic CO_2_ emissions for each arc segment (table S5) and converting to Tg C/year, with minimum to maximum ranges presented parenthetically, we find total average carbon emissions of 0.278 (0.105 to 0.450), 0.139 (0.023 to 0.256), and 0.038 (0.011 to 0.065) Tg C/year and for the eastern, central, and western Aleutian segments, respectively. When summed together, this yields a total Aleutian-Alaska volcanic output flux of 0.455 (0.140 to 0.771) Tg C/year (table S6). We note that the total arc-wide CO_2_ flux calculated here is nearly identical to that calculated by Fischer *et al.* (*27*), with the main difference being the distribution of CO_2_ emissions along the arc.

### Carbon cycling

We next calculate how much subducted slab carbon is recycled to the atmosphere via volcanism in the Aleutian-Alaska Arc. Because of the potential contribution of crustal carbon sources in the eastern Aleutian segment, we focus our calculations on the central and western Aleutian arc segments, which have negligible crustal carbon. If we ignore the potential contribution of mantle, then the percentage of subducted slab carbon recycled to arc volcanoes is simply the sum of the average central and western Aleutian volcanic carbon output fluxes divided by the sum of the average SED + AOC carbon input fluxes for the same region. We find that ~16% of carbon entering the Aleutian-Alaska trench is returned to the atmosphere via arc volcanism (table S6).

In the following sections, we estimate how much SED and AOC carbon is recycled to Aleutian-Alaska volcanoes on an arc segment scale. Because the δ^13^C composition of subducted sediments differs depending on the assumption of bulk versus incoming sediments being subducted to sub-arc depths, and the isotopic composition of SED controls the proportion of SED and AOC recycled, we must consider the isotopic composition and associated fluxes in the following calculations. We first calculate the average flux of SED and AOC carbon recycled to volcanoes within each arc segment by multiplying the arc segment–specific volcanic carbon fluxes by the average *f*_SED_ and *f*_AOC_ from our mixing models. As mentioned previously, we expect that the average of bulk and incoming sediments best represents how much SED is actually subducted. We therefore expect that recycling calculations done using proportions *f*_SED_ and *f*_AOC_ calculated using the carbon-He mixing model based on the minimum bulk sediment δ^13^C composition for each arc segment to be the most accurate. We refer to calculations done using these parameters as our “best estimate” for the following carbon cycling calculations. We also acknowledge the possibility that all trench-fill sediments are lost to shallow processes, such that only incoming sediments are subducted to sub-arc depths. Our results suggest that this scenario is likely for the western Aleutian sector. We therefore consider a scenario that uses the proportions of *f*_SED_ and *f*_AOC_ calculated using the carbon-isotope mixing model results for incoming sediments only (table S1 and fig. S4) and refer to this as the “end-member” scenario. Because the incoming sediment carbon composition is isotopically heavier than that of the bulk sediments, the resulting mixing proportions to explain volcanic outputs in the end-member scenario require more SED and less AOC than the best-estimate scenario described above (table S4). We expect actual carbon recycling on an arc-wide scale to fall between our best-estimate and end-member scenario results. Upper and lower flux bounds for both scenarios are calculated by multiplying the minimum and maximum values of *f*_SED_ and *f*_AOC_ (table S1) by the minimum and maximum flux of volcanic outputs (table S5). These results are summarized in table S6.

Last, we calculate the percentage of subducted SED and AOC carbon recycled to arc volcanoes for both the best-estimate and end-member scenarios. This calculation is simply the average output flux of SED and AOC (calculated in the previous section) divided by their respective average input flux. In the case of AOC, we assume that all subducted AOC carbon reaches sub-arc depths, such that the input flux for both scenarios is the same. In the case of SED, we use the average input flux of incoming and bulk sediments as the most likely SED flux for both the best-estimate and end-member scenarios. Upper and lower bounds (presented parenthetically in the following) for both scenarios are calculated by dividing the minimum output flux by the maximum input flux and vice versa. In the case of SED, we use the bulk sediment flux as the maximum SED input flux and the incoming sediment flux as the minimum possible SED input flux for both scenarios. We consider actual carbon recycling percentages to be best represented by the range in average values between the best-estimate and end-member scenarios and consider our absolute upper and lower boundaries as the minimum and maximum values observed from the two scenarios. These results can be found in table S6.

For the best-estimate scenario, we find that ~42.6% (3.2 to >100%) of SED-derived carbon and ~5.4% (0.3 to 23.0%) AOC-derived carbon are recycled to central Aleutian volcanoes. For this scenario for the western Aleutian segment, we find that no SED-derived carbon and ~6.2% (0.7 to 24.8%) of AOC-derived carbon are recycled to arc volcanoes. In the end-member scenario, we find that ~60.6% (4.9 to >100%) of SED carbon is recycled to central Aleutian volcanoes, with no AOC carbon recycled to central Aleutian volcanoes. For the western Aleutian segment, we find that ~6.4% (0 to >100%) of SED carbon and ~9.4% (1.4 to 33.5%) of AOC carbon are recycled to western Aleutian volcanoes.

Considering our best-estimate and end-member scenario results collectively, we can conclude that the most likely percentage of SED recycled to central Aleutian volcanoes is ~43 to 61%, with absolute upper and lower bounds of 3 to 100%, while the most likely percentage of AOC recycled to western Aleutian volcanoes is 6 to 9%, with absolute upper and lower bounds of 1 to 34% (table S6). Therefore, even considering the farthest extent of our mixing bounds and flux uncertainties, quantifiable recycling of SED to central Aleutian volcanoes and AOC to western Aleutian volcanoes is required to explain volcanic carbon outputs.
