# Supplementary material for: Tracking carbon from subduction to outgassing along the Aleutian-Alaska Volcanic Arc
Source: Sci Adv. 2023 Jun 28;9(26):eadf3024. doi: 10.1126/sciadv.adf3024 (PMC12488040; doi:10.1126/sciadv.adf3024)
Supplement: Supplementary file 1 — Figs. S1 to S4 Table S4 Legends for tables S1 to S3, S5 and S6 [file sciadv.adf3024_sm.pdf]

Science Advances
AAAS

Supplementary Materials for

Tracking carbon from subduction to outgassing along the Aleutian-Alaska Volcanic Arc

Taryn Lopez *et al.*

Corresponding author: Taryn Lopez, tmlopez@alaska.edu

Sci. Adv. **9**, eadf3024 (2023)
DOI: 10.1126/sciadv.adf3024

The PDF file includes:

Figs. S1 to S4
Table S4
Legends for tables S1 to S3, S5 and S6

Other Supplementary Material for this manuscript includes the following:

Tables S1 to S3, S5 and S6

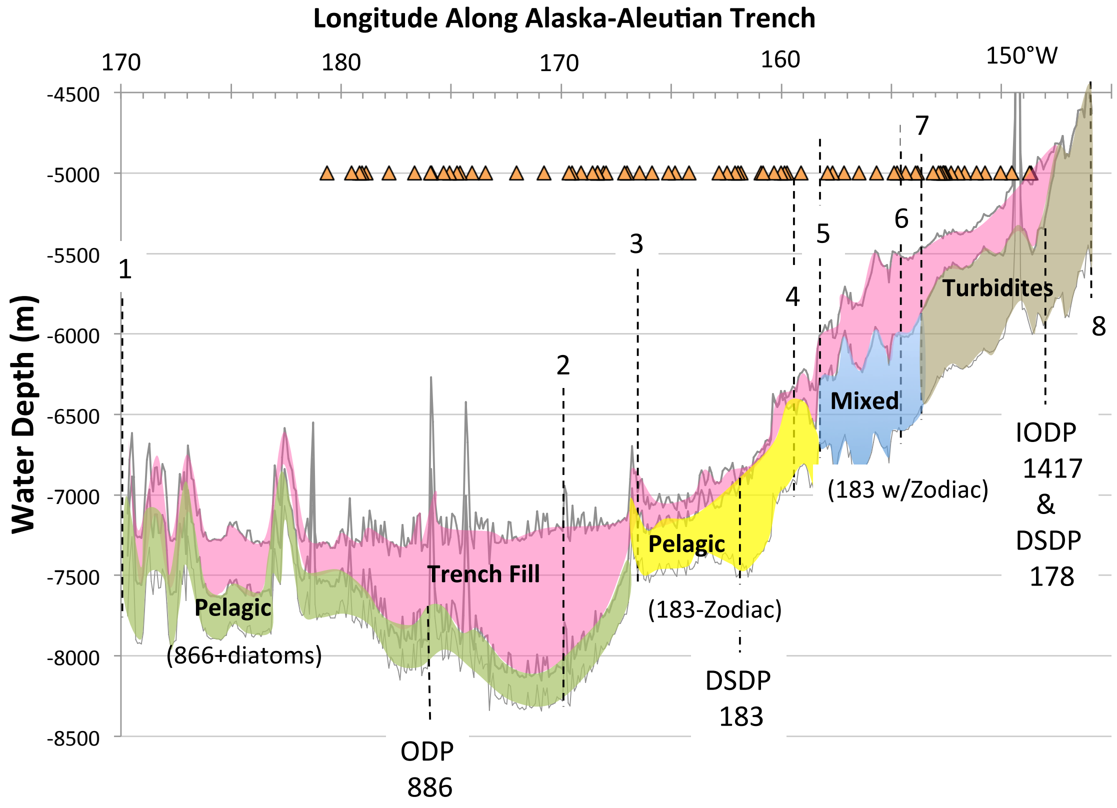

Fig. S1. Depth of the seafloor and thickness of sediments on the incoming Pacific plate and in the Aleutian-Alaska trench. Volcanoes of the arc plotted as orange triangles along the top of the diagram, at the projected trench longitude. Colored shading reflects different incoming marine sedimentary provinces, and the trench-fill (pink). Reference drill sites are labeled where they project to the trench, and guide calculation of carbon fluxes and isotopic compositions at the 8 nodes (labeled vertical dashed lines). Amlia Fracture Zone (~173°W) serves as a dam to downslope sediment transport in the trench axis.

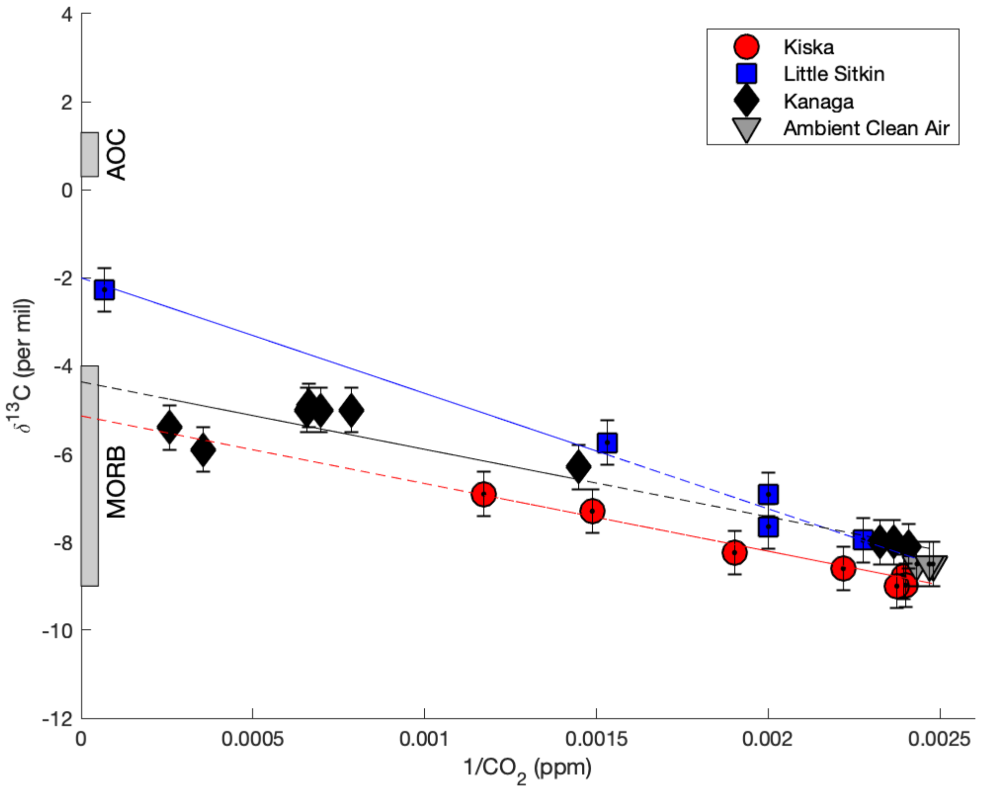

Fig. S2. 1/CO_2 and δ^{13}C-CO_2 compositions of volcanic and clean air samples. Samples of plume and ambient air at varying proportions collected for WA volcanoes and used to extrapolate to each volcano's magmatic CO_2 concentration and **δ**^{13}C-CO_2 composition.

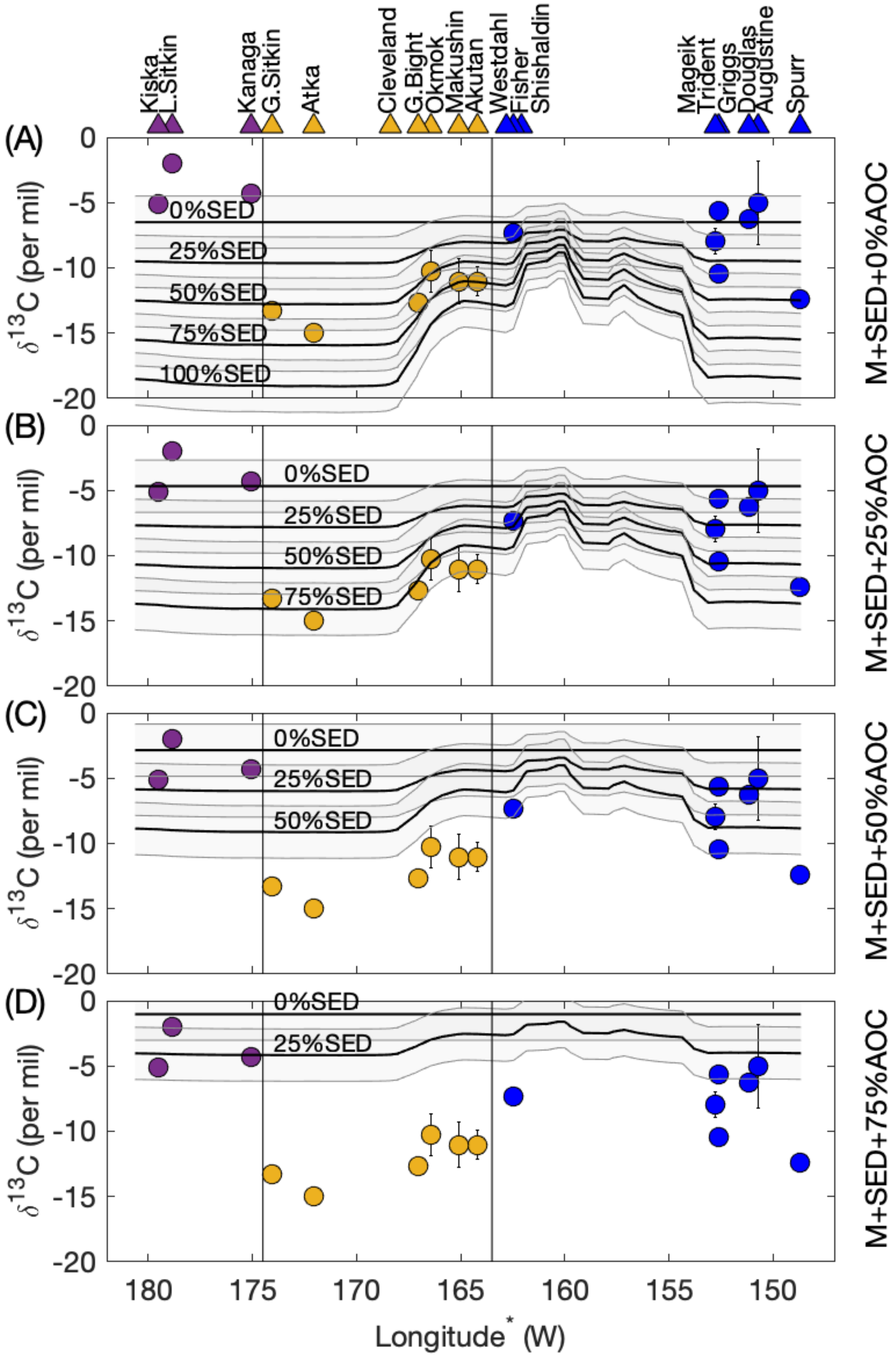

Fig. S3. C-isotope mixing model results. Results of the carbon-isotope model showing observed volcanic $\boldsymbol{\delta}^{13}C$-$CO_2$ outputs from fumarole samples (circles) and the predicted volcanic carbon outputs calculated from the model as mixtures of *M*, *SED* and *AOC* for four scenarios representing 0% *AOC* (A), 25% *AOC* (B), 50% *AOC* (C), and 75% *AOC* (D) are shown. Each line shows the

percent of carbon supplied from bulk sediment (*SED*) for that scenario, with the remaining carbon being attributed to mantle (*M*) and/or *AOC* sources, where 1 - *AOC* - *SED* = *M*. Shaded regions reflect ±2‰ uncertainties. As increasing amounts of *AOC* carbon are considered, *M* and *SED* percentages are systematically decreased (e.g., *M* + *SED* = 25% in D). Data are colored by arc segment, where blue, yellow, and purple represent eastern, central, and western Aleutians, respectively.

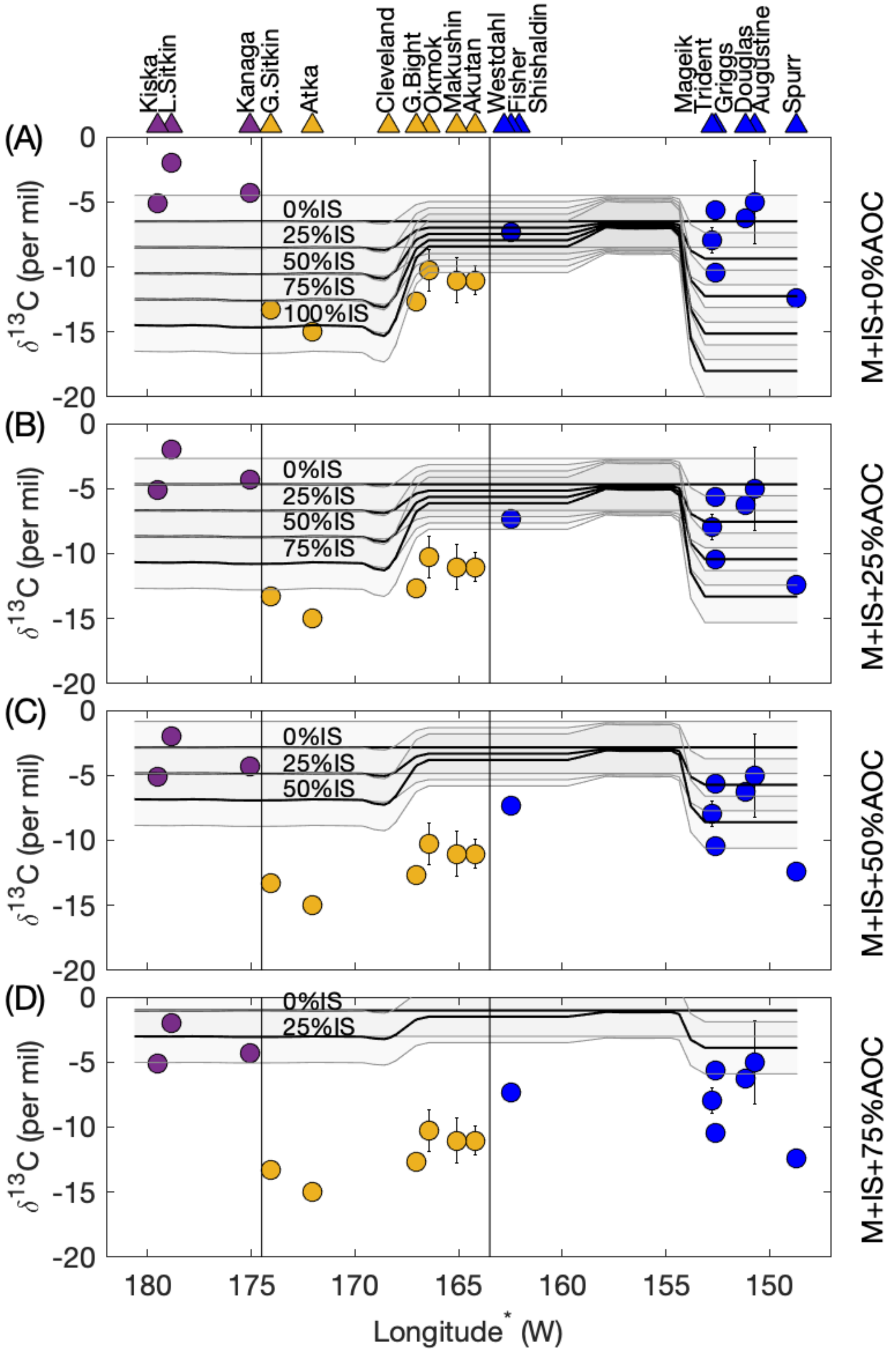

Fig. S4. C-isotope mixing model results for an incoming sediment source. Results of the carbon-isotope model showing observed volcanic **δ**[13]C-CO_2 outputs from fumarole samples (circles) and the predicted volcanic carbon outputs calculated as mixtures of mantle (*M*), incoming sediment only (*IS*) and *AOC* for four scenarios representing 0% *AOC* (A), 25% *AOC* (B), 50%

AOC (C), and 75% *AOC* (D). This figure is the same as Figure S2 but calculated using the isotopically heavier (higher) *IS* $\delta^{13}C$ end-member composition instead of the bulk sediment (*SED*) end-member. Each line shows the percent of *IS* for that scenario, with the remaining carbon being attributed to mantle and/or *AOC* sources (1 - *AOC* - *IS* = *M*), shaded regions reflect ±2 per mil uncertainties. As increasing amounts of *AOC* carbon are considered, *M* and *IS* proportions are systematically decreased (e.g., *M* + *IS* = 25% in D). Data are colored by arc segment, where blue, yellow, and purple represent eastern, central, and western Aleutians, respectively.

	Mantle	Carbonate (AOC)	Mantle + Carbonate	Sediment
Eastern Aleutians carbon-He	5-47%	37-49%	45-83%	16-55%
*Eastern Aleutians carbon-isotope – Incoming Sediment only	0-75%	0-75%	25-100%	0-75%
*Eastern Aleutians carbon-isotope – Bulk Sediment	0-50%	0-75%	50-100%	0-50%
Central Aleutians carbon-He	8-30%	15-29%	23-50%	50-77%
**Central Aleutians carbon-isotope – Incoming Sediment only	0-<25%	0%	0-<25%	>75-100%
**Central Aleutians carbon-isotope – Bulk Sediment	25-50%	0-25%	25-50%	50-75%
Western Aleutians carbon-He	53-73%	27-47%	100%	0%
*Western Aleutians carbon-isotope – Incoming Sediment only	0-50%	50-75%	75-100%	0-25%
*Western Aleutians carbon-isotope – Bulk Sediment	0-75%	25-75%	75-100%	0-25%

**Best fit to data for at least three volcanoes in this region.*
***Best fit to three lowest (lightest) $\delta^{13}C$ values*

Table S4. Summary of carbon mixing model results. Summary of the range in mean carbon source contributions to Aleutian-Alaska volcanoes by arc segment as inferred from carbon-He and carbon-isotope mixing models.

Table S1. (separate file)

Aleutian-Alaska subduction inputs. Characteristics of Aleutian-Alaska subduction parameters, sediment inputs, and carbon-isotope mixing model results.

Table S2. (separate file)

Aleutian-Alaska volcanic gas outputs. Chemical and isotope compositions of key gas species from Aleutian-Alaska volcanic gas outputs and carbon-He mixing results.

Table S3. (separate file)

Helium isotope ratios in fluid inclusions within olivine crystals.

Table S5. (separate file)

Aleutian-Alaska volcanic CO_2 outputs.

Table S6. (separate file)

Aleutian-Alaska carbon budget.
